# Abortion Experiences and Perspectives Amongst Migrants and Refugees: A Systematic Review

**DOI:** 10.3390/ijerph21030312

**Published:** 2024-03-08

**Authors:** Sharanya Napier-Raman, Syeda Zakia Hossain, Elias Mpofu, Mi-Joung Lee, Pranee Liamputtong, Tinashe Dune

**Affiliations:** 1 Sydney School of Health Sciences, Faculty of Medicine and Health, The University of Sydney, Sydney, NSW 2050, Australia; zakia.hossain@sydney.edu.au (S.Z.H.); elias.mpofu@sydney.edu.au (E.M.); mi-joung.lee@sydney.edu.au (M.-J.L.); 2 College of Health Sciences, VinUniversity, Gia Lam District, Hanoi 100000, Vietnam; pranee.l@vinuni.edu.vn; 3 Translational Health Research Institute, Western Sydney University, Campbeltown, NSW 2150, Australia; t.dune@westernsydney.edu.au

**Keywords:** abortion, migrants, refugees, reproductive rights, reproductive health

## Abstract

(1) Background: Access to abortion care is a crucial reproductive health right. Refugees and migrants may have restricted access to and utilisation of abortion care, associated with histories of displacement, precarious migrant and citizenship status and difficulty navigating unfamiliar host country healthcare systems. However, there is limited evidence on the abortion experiences and perspectives of refugees and migrants. Moreover, existing research has not been synthesised to identify trends informing sexual and reproductive care access among this marginalised population. This systematic review aimed to address this gap in the cumulative evidence on refugee and migrant experiences and perspectives of abortion in host countries. (2) Methods: Following the Preferred Reporting Items for Systematic Reviews and Meta-Analyses (PRISMA) guidelines, we searched the following databases for studies on refugee and migrant abortion attitudes, decision making and experiences: Embase, Medline, CINAHL, Web of Science, Sociological Abstracts, and Scopus. We also searched the grey literature on the same. Inclusion criteria specified qualitative studies involving migrant and/or refugee populations, examining their abortion experiences, attitudes or perspectives, written in English, published between January 2000 and December 2022. Two reviewers screened titles, abstracts and full-text articles, resulting in 27 articles included in the review, following consensus checks by two co-authors. The included studies were assessed for methodological quality using the Critical Appraisal Skills Programme tool. (3) Results: Abortion was stigmatised and generally considered impermissible and undesirable. However, participants discussed socioculturally determined ‘exceptions’ to this, positing circumstances where abortion was acceptable. There were striking differences in experiences between participants in higher-income settings and those in lower- and middle-income settings. Difficulties accessing care were ubiquitous but were heightened in lower-resource settings and among participants with precarious citizenship, financial and legal statuses. (4) Conclusions: The findings highlight the need for an international convention to guide policy and programming that acknowledges the specific abortion requirements of migrant and refugee communities, with attention to their financial, legal and social precarity.

## 1. Introduction

Access to abortion care is a reproductive right, linked to rights to health, privacy and bodily autonomy and freedom from cruel, inhumane, and degrading treatment. Comprehensive abortion care is essential to reproductive autonomy: the ability to control whether, when and how many children one has. Annually, between 2015 and 2019, there were an estimated 121 million unintended pregnancies globally, 61 per cent resulting in abortion [1]. Induced abortion—through medication or surgical procedures [2]—is a low-risk, simple intervention when performed safely [3]. Abortion is sought globally, irrespective of legal and structural restrictions. It remains, however, highly contested, stigmatised, and censored. Despite universal need, abortion experiences and understandings are not universal. Abortion trajectories involve the interrelationship between individuals’ abortion-specific experiences, individual context, and the regional, national and international context [4]. Intersecting socioecological factors including gender, race, class, immigration status, legal structures, and health access shape how individuals actualise their reproductive rights [5].

The abortion attitudes and experiences of migrants and refugees are likely influenced by factors that can be described using the socioecological model (SEM). These include individual-level factors such as education and beliefs, interpersonal-level factors of family and community networks, institutional-level factors relating to healthcare services, and societal-level factors of abortion legislation and migration policy. Migrants and refugees experience barriers to healthcare, particularly surrounding sexual and reproductive health (SRH) [6,7,8,9]. Displacement and migration can interrupt SRH education and care access. Moreover, in many migrant and refugee communities, taboos and stigma surround SRH [6,8,10], especially abortion [11,12]. Nevertheless, compared to women born in Australia and New Zealand, the United States, and Northern and Western Europe, immigrant women born in all world regions except Southern Africa were 2–5 times more likely to have an induced abortion [13]. Low SRH and contraceptive knowledge have been noted among migrant and refugee communities [7], which may increase the likelihood of unintended pregnancy and abortion. The SEM provides a framework for examining how abortion experiences, attitudes and decision-making among migrants and refugees are influenced by multiple factors across individual, interpersonal, institutional, and societal levels.

This review focuses on induced abortion. While past reviews have synthesised the decision-making, attitudes and experiences of abortion-seekers generally [14,15,16], research on the experiences and perspectives of migrants and refugees is not yet aggregated. Given the focus on experiences and perspectives, we considered qualitative research methods most appropriate. As such, this systematic review aims to synthesise qualitative research related to the induced abortion experiences and perspectives of migrants and refugees.

## 2. Materials and Methods

### 2.1. Search

**Figure 1:**
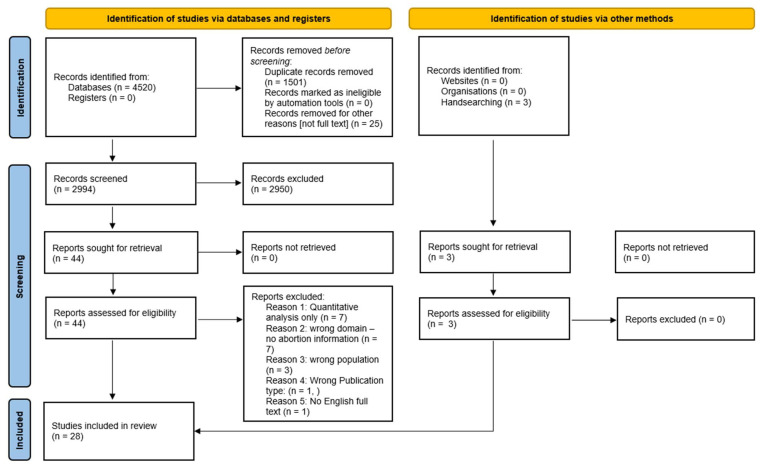
PRISMA flow diagram of search and study inclusion process [18].

A systematic review of qualitative literature was conducted in line with PRISMA guidelines (Figure 1). The review protocol was registered on PROSPERO: CRD42023480376. Key subject areas were searched across six databases (Embase, Medline, CINAHL, Web of Science, Sociological Abstracts, Scopus) in addition to Google Scholar searching, hand and grey-literature searches. Searches were limited to sources from the year 2000 onwards.

The original purpose of this review was to examine migrant and refugee youth perspectives. However, initial searches focusing on migrant and refugee youth yielded insufficient results, leading to revised scope and search terms. Thus, searches were conducted around two key subject areas: migrants and/or refugees, and abortion. Search results were uploaded to EndNote 21 and duplicates removed [17].

### 2.2. Study Selection

Due to broad search terms, the initial database search yielded large numbers (*n* = 4520) (Figure 1). After duplicates were removed, titles and abstracts were screened for inclusion by two reviewers. Discrepancies between reviewers were resolved through discussion. Post initial peer review, an additional study was added.

During initial title and abstract screening, studies that focused on sexual and reproductive health but did not necessarily mention abortion in titles or abstracts were included to prevent premature exclusion of potentially valuable studies. Non-English-language publications were excluded. The inclusion criteria and search strategy are outlined in Table 1. Studies of second-generation migrants were included in the selection criteria as they experience similar barriers to care and influences of social networks to first-generation migrants.

### 2.3. Quality Assessment

Included studies were independently assessed for methodological quality using the Critical Appraisal Skills Programme tool (CASP) Qualitative Studies Checklist [19]. A second reviewer verified quality assessment scoring; any disagreements were resolved through discussion. The CASP tool offers a simple, systematic means for evaluating a study’s validity and quality. The tool consists of ten questions assessing methodological rigour, relevance, and credibility of findings for which reviewers answer ‘yes’, ‘can’t tell’ or ‘no’. A quality score out of ten was calculated based on answers to each question, with one point awarded for ‘yes’ and none for ‘can’t tell’ or ‘no’ responses (Table 2).

### 2.4. Data Extraction and Analysis

Characteristics were extracted from all included studies (Table 1). Thematic synthesis methods were used to analyse findings [48]. Full-text studies were uploaded into QSR’s NVivo, version 12 software (Lumivero, Burlington, NJ, USA) for analysis [49]. Thematic synthesis was conducted in three stages, beginning with line-by-line coding of all text under the ‘findings’ or ‘results’ sections of studies. Descriptive themes were then developed by grouping codes based on similarity. Finally, descriptive themes were developed into analytical themes. Following initial data extraction and analysis by one reviewer, the findings were reviewed and verified by the review team. A model of the key findings, mapped onto the levels of the SEM, is presented alongside the results.

#### A Note on Gender

While we acknowledge that abortion-seekers may not identify as female, all studies in this review identified participants who underwent abortion as female. The results and discussion of this paper will thus refer to migrant and refugee women when referring to participants who had abortions.

## 3. Results

### 3.1. Participants’ Characteristics

A total of 28 studies were included in the final review. Study characteristics and quality scores are detailed in Table 2. Most studies (*n* = 17) were conducted in high-income settings; ten were in middle-income settings and one was in a low-income setting [50]. Where possible, details on participants’ migrant status have been noted (Table 2), but these were not provided in all studies. Fourteen studies identified participants as ‘migrants’ (three of which included first- and second-generation migrants, though study results did not distinguish between these groups); five studies focused on ‘refugees’; seven studies included an unspecified combination of migrants/refugees/cross-border migrants; one study included documented and undocumented migrants and another illegal immigrants. The ethnographic nature of some studies meant detail on sample sizes and full participant demographics were absent. A significant proportion of studies (*n* = 9) were conducted along the Thai border and in the United States (*n* = 6). Studies included a range of participant backgrounds, though participant groups from Asia (*n* = 14), specifically Burma/Myanmar (*n* = 9), were most represented. As per the Center for Reproductive Rights classification system [47], fifteen studies were conducted in settings where abortion laws were most permissive, being in Category 5, “permitted on request”. Two studies were conducted in Category 4 settings, where abortion is legal on “broad social or economic grounds”. Nine studies were in Category 3 settings, where abortion is legal only “to preserve health”, and two were conducted in a Category 2 setting, where abortion is legal only to “save a woman’s life”. No studies were from Category 1 settings, where abortion is “prohibited altogether”.

**Figure 2:**
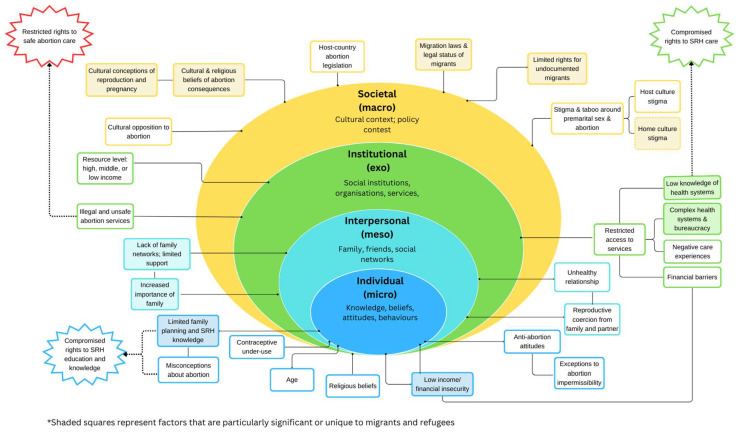
Key findings across socioecological levels and corresponding sexual and reproductive rights.

Not all studies focused specifically on abortion, while some focused on specific types, methods and reasons for abortion. Termination of pregnancy for genetic anomalies was the focus of three studies [20,30,31], and sex-selective abortion the focus of two [38,45]. While a few studies mentioned abortion-seekers’ SRH rights [24,29,41], only Deeb-Sossa and Billings included an extensive examination of rights, using a reproductive justice lens [26]. No studies explicitly used a rights framework to examine migrant and refugee abortion experiences, attitudes and/or perspectives. The following section presents results under themes identified through the data analysis. Figure 2 summarises key findings and maps them onto the SEM, illustrating how findings span across socioecological levels and how these relate to key SRH rights.

### 3.2. Attitudes, Perceptions and Beliefs towards Abortion and Abortion Permissibility

#### 3.2.1. Attitudes and Perceptions

Attitudes towards abortion among migrants and refugees were diverse and nuanced, often holding contradictions between anti-abortion moral stances and lived realities. Perspectives ranged from strongly anti-abortion [28], to pro-choice, though only in one study did participants firmly hold pro-abortion stances [39]. Regardless of whether participants were themselves anti-abortion, sociocultural stigma and taboo were mentioned in all studies. In most studies, there were participants who believed abortion was wrong, immoral or impermissible [11,20,22,23,28,30,31,33,34,35,39,40,41,44,46], even those who themselves accessed abortion care.

Participants from diverse backgrounds and settlement locations viewed abortion negatively [28,31,34,40], describing it as ‘very ugly’ [40] and a sign of a ‘bad heart’ [35]. Some participants perceived abortion as an unforgivable transgression, wrong to even consider [28,41]. As one Somali woman asserted, ‘we don’t even know anything about abortion’ [41]. Frequently, the strongest anti-abortion stances were tied to religiosity, with termination considered a ‘sin’ in multiple studies [28,33,40,41,46]. Imagery of murder or killing was used to describe abortion [31,35,40,46]. In some cases, foetal agency was asserted, with conception considered an indication that a child wants to live [35]. Participants asserted future punishment would befall those who terminated [22,30,35,40], ranging from future sterility or sickness for the abortion-seeker, illness in future children, to indeterminate punishment by God. In one study, participants believed abortion-seekers would be punished in their next life by having ‘to eat back the things she aborted in a previous life’ [22]. Foetal agency was again invoked, with claims the aborted baby would inflict punishment upon the person who conceived them [35]. Despite broad negative perceptions, it was admitted that abortion was common but never discussed [28,38,39,40].

#### 3.2.2. Knowledge and Beliefs Surrounding Abortion and Family Planning

Participants held a range of knowledge and beliefs surrounding abortion and broader SRH, often informed by cultural, social and contextual factors. Low contraceptive awareness and limited SRH knowledge were pervasive [22,27,28,29,32,33,34,40,41,43,44,45,46]; participants themselves attributed unplanned pregnancy and abortion in their communities to these deficits [28,40,43,46] (see Figure 2). Religious participants believed family ‘planning’ was a fallacy: Muslim Somali, Moroccan and Turkish women, and Christian and Catholic Haitians and Lusophone African women asserted pregnancy should not be controlled [28,30,31,40,41]. In one study, the emergency contraceptive pill was considered abortion and thus impermissible [40]. For some, abortion was considered contraception [24,32]: ‘abortion is the contraceptive choice and birth control method we can use’ [32].

Some migrants and refugees held culturally informed understandings of pregnancy and abortion. Burmese women believed pregnancy commences when the foetus begins moving like a ‘jumping shrimp’ [23]. Pregnancy termination before this stage was considered a means of treating amenorrhea rather than actual abortion. There were different understandings of when abortion could be performed. Some believed abortions could be performed at any stage before birth [32], while others asserted that abortion could only be performed in the first trimester [35], the first 40 days [31], or before 5 months’ gestation [35]. There were also beliefs that multiple abortions—even safe, clinically provided medical abortions—would ‘damage the womb’ [44]. While studies reported on misconceptions around contraceptives and cultural conceptions of reproduction, there was little on what participants knew about formal abortion procedures.

#### 3.2.3. Abortion Permissibility

Abortion permissibility varied, particularly as participants navigated tensions between anti-abortion values and the necessity of abortion in their lived realities. Generally, abortion was presented as generally impermissible, but with circumstantial exceptions—both by abortion-seekers and broader communities. Burmese women seeking abortion care described abortion as for ‘those who do not have a beautiful life’ [44]: acceptable if parent(s) cannot care for children [33,43]. In studies examining foetal anomaly testing and termination, participants believed certain disabilities and genetic conditions made abortion permissible [20,30,31]. Some participants, however, asserted ‘a child has a right to live, no matter what condition’ [30,31], reframing disability as a ‘test’ from God. Abortion to save the mother’s life was mentioned in three studies [30,31,35]. Muslim women in Gitsel-van der Wal et al.’s studies asserted Islam permitted abortion in rape cases [30,31]. In Liamputtong’s study, Hmong women had cultural beliefs about abortion as a right earned by women of a certain age—acceptable for older women who have already had ‘enough’ children [35]. Participants also identified time frames in which an abortion was permissible [26,31], Mexican and Burmese women similarly viewing the foetus in early pregnancy as ‘no baby’, only ‘blood’ and not yet ‘human’ [23,26]. Thus, despite stigma in all studies, very few migrants and refugees considered abortion always, irrefutably wrong.

### 3.3. Decision Making

#### 3.3.1. Financial/Economic Factors

While migrants’ and refugees’ reasons for seeking abortion were diverse, socioeconomic strain and poverty consistently shaped decisions [23,24,33,39,40,42,43,44,45,46] (Figure 2). Financial burdens of childrearing and pregnancy costs were insurmountable, especially when participants already had children. Under- and unemployment were common. Participants endured precarious, often unregulated work conditions that were not conducive to childrearing, or had to work constantly to survive, leaving no time for childcare [21,39,42,43,44]. This was exacerbated for illegal or undocumented migrants, who were not entitled to governmental support or worker’s rights [23,24,42]. Participants described employers coercing them into undergoing abortion [23,24,32,43], threatening deportation, denying work permits [23,24], and firing them for becoming pregnant [40]. Conversely, in Royer et al.’s study, Congolese refugees described children as an ‘investment’ rather than a financial burden, citing the potential future benefit of a child as a reason to not abort [41].

For many participants, the financial strain of childrearing was compounded by the precarity of their lived reality. The loss of social networks brought about by migration was a crucial factor contributing to decision making. Participants were unable to afford childcare in host countries and lacked the social and familial networks that would typically provide parental support and care [28,40,41]. In one study, social support workers forced undocumented migrants to undergo abortion, with women’s illegal status leaving them unable to refuse such demands [42].

Life stage also influenced abortion decision making. In studies of young participants, age and lack of preparedness and maturity were frequently mentioned as reasons to seek abortion [11,22,46]. This was also the case for younger participants in some studies with broader participant age groups [21,26].

#### 3.3.2. Sociocultural Factors

Decision making was also influenced by sociocultural factors and social pressure. Values of chastity and stigma around premarital sex were mentioned in multiple studies [11,29,32,35,46]. Despite abortion being similarly stigmatised in cultures and communities that held these values, abortion allowed participants to escape the social consequences of nonmarital pregnancy [11,27,46]. Abortion was a way to avoid ostracism and stigma brought by premarital pregnancy, and as such was often encouraged by families to save face [11,27,46]. Some participants described family pressure to abort [11,23,27,38,45,46], or they ended pregnancies to avoid disappointing families or to continue fulfilling familial caregiving responsibilities [11,26]. Others asserted that relatives would pressure them not to abort [46]. Some migrant and refugee women were pressured into abortion by partners, who were frequently women’s sole financial and social support in their host countries [38,39,42,45]. Moreover, unstable relationships and domestic abuse were key reasons for abortion [21,23,24,39,43]. Indian women in studies of sex-selective abortion, and Palestinian women in Gaza, described being physically and emotionally abused and threatened by husbands and in-laws for having female children, which informed decisions to terminate based on sex [25,38,45]. Sex-selective abortion was also tied to sociocultural gender norms valuing male children as breadwinners over female children [38,45].

#### 3.3.3. Contraceptive Failure and Under-Use

Under-use of contraceptives significantly contributed to unintended pregnancy and, subsequently, abortion decisions. As illustrated in Figure 2, inadequate family planning knowledge was a common reason for contraceptive non-use [22,23,24,28,29,32,42]. Misconceptions, particularly beliefs that contraceptives cause infertility [22,32,34,42], and stigma associating premarital contraceptive use with prostitution and promiscuity served as barriers to family planning [22,32,33]. Sexual assault, coercion and forced unprotected sex were common reasons for unintended pregnancy resulting in abortion [21,23,24,32,34,41,42]. Many women could not afford regular contraceptives [23,33,34,42] and faced supply limitations, restricted access to services, and risk of deportation and detention when travelling to procure contraceptives [23,29,32,33,34,42]. This lack of contraceptive options rendered abortion one of the only viable methods for managing fertility.

### 3.4. Accessing Abortion Care

Access to and experiences with abortion care were influenced by factors including immigration and legal status, employment and socioeconomic circumstances, and the legislature and health systems of host countries. Abortion accessibility varied significantly across studies, particularly access to safe, facility-based care. Studies from high-income countries (HICs) that discussed abortion access focused on the difficulty migrant and refugee participants had in navigating health systems and receiving formal care. Contrastingly, studies in middle- and low-income settings focused on informal and unsafe abortion methods or reported on non-profit programs to mitigate these practices. While participants in high-income countries faced barriers to accessing care, those in middle- and low-income countries (LMICs) frequently had no care to access.

#### 3.4.1. Accessing Formal Care

Across all studies examining access to abortion, migrants and refugees faced barriers. Abortion-seekers navigated complex, unfamiliar health systems with little support. Participants in numerous studies lacked knowledge of how to access care, were unaware of entitlements they had to care, and found the health systems of host countries confusing [21,26,36,37,42,43] (Figure 2). In two studies, participants mistakenly believed abortion was illegal in their host countries [11,28]. Participants in another study believed male partner consent was required for abortion [27]. Accessing services often required multiple steps and appointments [21,26,37]. Due to her age, one 17-year-old Mexican immigrant in North Carolina was required to obtain a judicial bypass to access care, a costly and complicated process requiring the abortion-seeker to be interrogated by a judge [26]. Those who were able to access care often faced delays [21,36,37]. Language barriers and unfamiliarity with host country healthcare and legislature compounded these issues, as did financial strain and precarious legal and citizenship status. No studies mentioned decision making between medication or procedural abortion; women accessed whatever care was available to them, particularly when they were beneficiaries of subsidised, safe abortion programs [21,37,43,44]. There was little discussion of self-managed abortion outside unsafe abortion methods. Formal, self-managed abortion was only specifically examined in one study reporting on a community-based misoprostol distribution program along the Thai border [44].

#### 3.4.2. Healthcare Experiences

For those who accessed healthcare, experiences were varied. Participants in high- and lower-income settings described being shouted at, chastised, treated roughly and misinformed by healthcare workers [21,26,36,37]. One Congolese woman seeking post-abortion care believed nurses intentionally made her wait, bleeding for hours, as punishment for self-inducing her abortion [36]. However, some migrants and refugees reported positive experiences, particularly those who received care from non-profit safe abortion programs established along the Thai–Burma border [21,43,44]. Studies examining the success of such programs found participants had overwhelmingly positive responses and advocated for the expansion of free safe abortion care [21,43,44]. The significance of such programs is further highlighted by the prevalence of unsafe abortion methods found by this review, which almost exclusively occurred in LMICs and under-privileged settings [21,23,24,27].

#### 3.4.3. Barriers to Accessing Care—Financial Barriers

Financial barriers to care were common [11,23,32,39,43,44]. The extent of financial burdens varied, from participants in HICs being unable to afford preferred private care [39] to illegal immigrants in LMICs being forced to perform unsafe self-induced abortions [23,24,32]. Some participants were unable to afford transport to receive care, while others could not afford pregnancy tests [36,43,44]. Additionally, undocumented migrants were barred from formal care and feared being deported, bribed, or arrested by authorities at clinics [21,23,24,26,43,44].

#### 3.4.4. Unregulated Abortions

This review found a vast array of unregulated and ‘folk’ abortion methods used by migrants and refugees. Informal abortion methods comprised abdominal massage/physical manipulations, ingestion, insertion, or a combination of methods. Pummelling abortions were described by Hmong and Burmese migrants as vigorous, often painful, abdominal massage by lay abortionists [23,24,29,32,35]. Some participants described self-inducement attempts by beating their abdomens [32]. Physical exertion, including lifting weights and long hours of physically taxing labour, was another method commonly used by Southeast Asian migrants [23,24,29,32,35].

A range of abortifacients were described across studies, most commonly herbal concoctions. In some cases, as with Mexican and Hmong participants, these methods were deeply specific, provided by traditional healers to certain women at particular stages of pregnancy [26,35]. Other herbal remedies were less regulated. Burmese participants in Thailand ingested unlabelled herbal concoctions or used the ubiquitous ‘multipurpose’ ‘blood purifier’ Kathy Pan [23,24,32]. Participants also ingested large doses of contraceptive pills [32,36,40]. Congolese refugees in Uganda reported practices of ingesting detergent and crushed bottles [36]. Using alcohol as an abortifacient was discussed in three studies [23,24,26,40]. Self-managed abortion using legitimate but illegally obtained abortion medications was common [21,24,26,33], though often, women incorrectly administered pills, receiving no instructions and variable doses.

Stick abortions were the most common insertion abortion method [23,24,26,32,44]. Additionally, flower stems [23], chicken quills [23], bottles [36], blades and sharp instruments [32,36] were inserted into the vagina.

Seeking lay abortionists—untrained midwives, traditional healers and medicine women—was common in multiple studies. In Liamputtong’s study of Hmong women, traditional medicine women had extensive training and expertise to perform abortions and would only practice under specific conditions [35]. However, most non-professional abortions lacked regulation [21,23,24,29,43,44]. These forms of unregulated abortion were excruciatingly painful—both during and after the procedure—and potentially fatal [23,24,29,44]. Participants frequently spent all their savings on unsafe abortions [23,24]. In numerous cases, participants who underwent such procedures experienced severe illness and injury—including punctured bowel, uterus or bladders, incessant bleeding, blood clotting, permanent infertility, infection, and fevers [23,24,32,36].

Unregulated abortions often resulted in hospitalisation [23,24,32]. Accessing post-abortion care often meant significant financial burden [21,23] and navigating legal restrictions [36].

Participants underwent unregulated abortions for myriad reasons. In Deeb-Sossa and Liamputtong’s studies [26,35], traditional healers and folk methods were a ‘first resort’ [35]. However, for refugee and undocumented migrants across the Thai border and in Uganda, unregulated and self-induced abortions were the only option [32,36,43]. For these participants, lack of documentation and financial stability, as well as macro-level legal restrictions on abortion (Figure 2), made formal procedures financially and legally unviable. Stigma around SRH and abortion also meant the covert nature of unsafe abortion was desirable [32,36]. Social and internalised stigma precluded information and service access and fostered secrecy, which led to unsafe, clandestine abortions.

## 4. Discussion

This review explicates the abortion attitudes, decision making and experiences of migrants and refugees, finding striking differences in experiences across lower- and higher-income settings. In LMICs, access to general reproductive care, knowledge and necessities, and access to safe abortion, was severely curtailed. This was compounded by precarious citizenship: many women in LMICs lacked legal migrant status, lived in camps and endured unstable, informal labour settings. While abortion is often lauded as a symbol of reproductive freedom, the experiences and attitudes of participants in this review provide a more nuanced depiction of abortion and ‘choice’. The intersections of gender, race, migration status, class and precarity make discourses of ‘choice’ inadequate for explaining the abortion attitudes and experiences of migrants and refugees. A reproductive justice approach, which acknowledges rights to (a) not have children, (b) have children, and (c) to parent children in healthy, safe environments, allows for the complexity of migrant and refugee abortion experiences [5]. These findings emphasise the importance of a rights-based, reproductive justice approach: an understanding of the ways ‘compounding injustices’ emergent from ‘societal, institutional and systemic contexts’ shape decision making and autonomy [9].

### 4.1. Attitudes and Beliefs

While migrants and refugees in this review often held negative attitudes towards abortion, the strength of these attitudes varied. Attitudes were shaped by individual-level factors including religious beliefs and SRH knowledge as well as macro-level cultural factors (Figure 2). Cultural and religious proscriptions against abortion were nuanced. Despite abortion being considered wrong, shameful or undesirable by most participants, there were a range of ‘exceptions’ to this, making the immorality of abortion context-dependent. This situational acceptability of abortion suggests that, rather than uncritically adhering to religious and sociocultural stricture, migrants and refugees actively negotiate values and attitudes. By adding situational exceptions to anti-abortion stances, participants managed the contradictions between sociocultural values and lived realities.

The strictest anti-abortion stances were rooted in religiosity, particularly Christian and Islamic doctrines. At the most extreme, this manifested in purposeful ignorance of abortion among Christian Sudanese women. Intentional ignorance has similarly been reported in young Muslim migrant women’s SRH attitudes, where ignorance becomes a method of maintaining purity [51]. In this review, ‘not knowing’ about abortion was a way to deny its existence. In many studies, there was tension between abortion taboos and attitudes, and actuality: abortion was stigmatised and never discussed, yet still commonly practised. Secrecy is a common manifestation of abortion stigma [52]. Moreover, many migrant and refugee communities maintain secrecy, silence and shame around SRH generally [7,10]. The taboos around abortion and the seeming contradictions between outward expression and actual practices seen in this review are therefore unsurprising.

Attitudes towards abortion were also shaped by societal (macro)-level cultural conceptions of reproductive health, including cultural understandings of foetal development and notions of foetal ‘agency’. We found a number of culturally specific consequences of abortion, particularly around future punishment by the aborted foetus or God. Furthermore, cultural conceptions of development provided participants with guidelines on the acceptable timespan in which a foetus could be aborted. Understanding these specific cultural constructions is crucial for improving care for migrant and refugee populations in settlement countries.

### 4.2. Knowledge

Previous research has shown that migrant and refugee populations often have inadequate SRH knowledge, attributed to limited SRH education in home countries, disruption of schooling, and stigma surrounding SRH within communities [7]. In this review, restricted discourse around SRH and lack of knowledge indicate that women’s agency and ability to make informed abortion decisions are impeded. Inadequate knowledge about unsafe abortion risks has serious implications for health and mortality. While no studies explicitly examined knowledge around medication abortion, illegally obtained, legitimate medication was used without any knowledge or instruction on administration. There were limited data on self-managed abortion in studies beyond unsafe abortion, which may indicate a lack of knowledge of medication abortion or limited access. Misinformation on abortion has been shown to delay abortion care trajectories [4,53] and lead to accessing unsafe abortion care [54]; Pagoto et al. describe an emerging abortion ‘infodemic’ where increasingly present misinformation exacerbates maternal mortality and targets the most vulnerable populations [55].

Research among non-migrant populations has indicated that women have limited knowledge of abortion legality and national legislation, even in countries with permissive laws [56]. Similarly, in this review, participants had low knowledge of abortion laws and health systems while facing the additional barrier of precarious citizenship and limited understanding of legal rights in host countries. Restricted rights to make informed choices were compounded by a lack of available, affordable contraceptive methods and SRH services, at times leading women to rely on abortion as a contraceptive method.

### 4.3. Decision Making

As with research on non-migrant populations, abortion decision making among migrant and refugee women was multifaceted [14]. Socioeconomic strain, however, is a primary reason for abortion decisions among non-migrants [14,16,54], which was also the case in this review. In their review of women’s abortion experiences, Lie et al. found the importance of pragmatism in decision making, with choices regarding termination and method influenced by financial considerations and perceptions of efficacy [15]. The significance of pragmatic decision making is similar in this review: participants made decisions around material conditions such as impoverishment or lack of support. Additionally, this pragmatism was seen in multiple studies where abortion was considered morally worse than non-marital pregnancy, yet preferable because the social shame and stigma of non-marital pregnancy could be avoided by a secret abortion. Participants’ ability to make pragmatic decisions, however, was often limited by material and social conditions. Undocumented and illegal immigrants did not have the option to pragmatically weigh up different abortion methods, unlike the participants in high-income countries in Lie et al.’s study. In this review, legal and financial restrictions meant some participants could only access clandestine, cheap, often unsafe terminations.

Research in LMICs has illustrated the importance of interpersonal-level factors of social networks, particularly partners and families, in abortion decision making [53]. More broadly, much existing literature highlights the importance of social influences on SRH decision making and experiences among migrant and refugee communities, with migrant and refugee conceptions of SRH incorporating notions of social risk alongside more conventional biomedical risk [6,7]. This strengthened presence of social ties is similarly evident in the abortion attitudes among internally displaced people living in camps or settlements [57,58]. Likewise, in this review, social stigma, partner coercion and family pressure were important abortion decision-making factors.

However, we also found that a lack of social support significantly shaped abortion decisions, with women being unable to care for children without family and social networks (Figure 2). Social isolation combined with financial dependency restricted women’s ability to counter partner coercion. In cases of sex-selective abortion, being cut off from birth families made it harder for women to counter coercion from in-laws and husbands. Social relationships take on heightened importance in settlement contexts where displacement has destroyed and disrupted home-country networks [7]. The disruption of social networks leaves parents without resources to care for and afford children while also intensifying the importance and influence of ‘re-created’ networks in host countries, increasing susceptibility to coercion and the power of social stigma. Relative social support and isolation undoubtedly vary depending on whether individuals live in ethnically homogenous communities (i.e., refugee camps) or are transient migrants or separated from others from their original communities. Our findings suggest a tension between the strong social cohesion created by migration and displacement and the loss of key home-country supports. Abortion decision making must thus be understood as situated in social context, informed both by pressure from social networks and by pressures experienced because of a lack of social network.

This review clearly illustrates how institutional- and societal-level factors shape abortion experiences (Figure 2). Decision making and agency were significantly curtailed by the lived experiences of migrant and refugee women. There were vast differences between populations in low-income and high-income nations. Those in conditions of heightened precarity—women in refugee camps, undocumented and temporary migrants—were barred from accessing safe abortions and were more likely to self-induce or seek unregulated, health-threatening abortions and experience serious post-abortion harm. Women’s ability to make abortion choices freely and autonomously was restricted by precarity. In Schoevers et al.’s study, illegal immigrants were forced into abortions by service providers [42]. Similarly, in the studies of migrant women living along the Thai border [23,24,32,43,44], illegality forced abortion decisions: women feared deportation and employment loss, which shaped decisions both to undergo abortion and use illicit methods. Crucially, however, participants in Schoevers et al.’s study resided in a high-income country and had access to safe, legal abortions via the Dutch healthcare system. Participants in studies along the Thai border, and those in refugee camps in Uganda, had no such access. Instead, women in these contexts opted for what the WHO would classify as ‘unsafe abortions’: terminations performed by ‘individuals lacking the necessary skills’, and/or in environments that fail to meet ‘minimal medical standards’ [3]. Though folk and traditional abortion methods by lay abortionists were discussed in two studies in high-income settings, only participants in low-income settings reported actual experiences with unsafe abortion as per the WHO definition. Given that 97% of unsafe abortions occur in LMICs [53], these findings are unsurprising.

It is important, however, to consider the social, economic and legal components that influence abortion decision making. An unregistered medically unsafe abortion may be considered a ‘safer’ legal or financial option for an illegal immigrant, allowing them to avoid job loss, deportation and punishment. Moreover, clandestine abortions may protect individuals from social stigma, ostracism and shaming that may come from non-marital pregnancy and abortion, thereby being socially ‘safer’. Multifaceted understandings of risk and safety that include financial, legal and social aspects are thus essential to ensure that safe abortion programs can effectively support migrants and refugees.

Despite finding significant differences in abortion experiences between participants in HICs and LMICs, across high- and low-income settings, migrants and refugees consistently faced barriers to care and had negative care experiences. Even in studies where participants could legally access abortion, navigating health systems was arduous [26,37,39]. Negative experiences with healthcare workers were similarly ubiquitous, irrespective of the host country’s abortion legislature or income level. Financial concerns were relevant to participants accessing abortion care across this review, revealing the significance of socioeconomic issues in both informing abortion decision making and determining care access.

The SEM provides a structure for understanding how various individual and structural factors shape experiences and attitudes towards abortion among migrants and refugees. Moreover, these factors have implications on numerous SRH rights, not simply abortion rights (see Figure 2). Our findings, illustrated in Figure 2, indicate that to create transformative change, reproductive justice interventions for migrants and refugees must address factors across all socioecological levels.

### 4.4. Limitations

Generally, this review is limited by the paucity of research on migrant and refugee abortion. We intentionally excluded internal migrants or internally displaced people (IDPs), as this would have too greatly expanded the study scope. However, for comprehensive understanding of abortion across population groups, future studies should examine how the experiences of internally displaced people in humanitarian settings compare to international migrants and refugees.

Broad inclusion criteria allowed for a larger set of studies and thus a more holistic view of migrant and refugee communities’ abortion attitudes, decision making and experiences. However, this meant some studies solely provided data on attitudes and potential decision-making factors, not the perspectives of actual abortion-seekers. There was an uneven distribution of study settings and ethnicity of participant groups: disproportionately, the included studies were from Asia, and the majority of those included participants from Southeast Asia. There was a noted scarcity of data from Africa and Latin America, perhaps due to the focus on international migrants/refugees. Some included studies examined only specific reasons for abortion, making the comparison of decision-making factors difficult. Many studies lacked thorough demographic reporting; there were no specific findings related to different generations of migrants (first, second or 1.5). Moreover, further research on age groups such as youth, who have specific needs and vulnerabilities, is necessary. There was limited detail on medication abortion in the included studies.

No included studies were conducted in countries in which abortion is completely prohibited, preventing a comprehensive view of how the most restrictive contexts shape abortion attitudes, decision making and experiences [47]. Moreover, in many study settings, abortion legislation has changed since the time of data collection, which may influence the current experiences of migrants and refugees [47]. Whether the introduction of permissive laws in Thailand has improved access to abortion and the experiences of migrant abortion-seekers must further be investigated. Conversely, examining the impact of post-*Roe* restrictions on vulnerable migrant and refugee groups in the United States is crucial.

Finally, there is a need for explicitly rights-based research, which was lacking in this review. While studies of abortion may implicitly address reproductive rights, further research that centres SRH rights, examining the ways migrants and refugees understand and actualise these rights, is needed.

## 5. Conclusions

Abortion attitudes and experiences among migrants and refugees are complex, informed by factors across all socioecological levels. Despite negative attitudes towards abortion apparent across this review, these attitudes were nuanced and malleable. Migrants and refugees frequently negotiated contradictions between anti-abortion moral stances and lived realities, asserting the conditional acceptability of abortion. Decision making was similarly multifaceted, though often dominated by financial concerns and social and relational influence. Cultural and social understandings of health, reproduction and pregnancy informed attitudes and decision making, and they must be acknowledged and accounted for in mainstream health provision.

Regardless of settlement location and residency status, migrants and refugees faced barriers to accessing abortion care. However, the intersections of poverty and immigration status significantly altered the extent and extremity of these barriers. This review indicates a clear need for better SRH education, information, care and support for migrants and refugees across settlement contexts. This includes improving health system literacy. Ensuring all migrants and refugees—including those in precarious citizenship, legal and financial situations—have access to safe, affordable abortion care is crucial. Moreover, a reproductive justice lens necessitates that migrants and refugees not only have abortion rights but also the right to have children and raise them in safe, healthy environments. Future health policies and programming must acknowledge the specific abortion and reproductive needs and experiences of migrant and refugee communities and the heightened vulnerability that emerges from financial, legal and social precarity.

## Figures and Tables

**Table 1:** Inclusion criteria and search terms.

Parameters	Inclusion	Exclusion	Key Terms/Strategy
Location	Global		
Language	English	Non-English	English only selected
Date	Published January 2000–December 2022	Published before 2000	Date restrictions: 1 January 2000–
Population	Studies including migrant and/or refugee populations; second-generation migrants; international students	Predominantly non-migrant/refugee study populations; internally displaced people; domestic migrants; service providers	Migrant* OR refugee* OR immigrant* OR ‘asylum seeker*’ OR ‘ethnic minorit*’ AND
Outcome/domain	Studies examining migrant and refugee abortion experiences, attitudes and/or perspectives; broader studies examining migrant and refugee SRH attitudes and/or experiences that include data on abortion	Studies examining non-migrant perspectives; Studies not examining participant perspectives or experiences	Abortion OR termination OR ‘termination of pregnancy’ OR ‘induced abortion’ OR ‘unplanned pregnancy’
Study design	Primary qualitative studies; grey literature	Quantitative studies; Abstract-only studies, reviews, opinion pieces	

* Boolean search terms (asterisk acts as truncator).

**Table 2:** Study characteristics.

Author (Year)	Data Collection Method	Setting	Setting Income Level	Abortion Legality *	Outcome/Domain	Relevant Sample Size *	Participant Residency/Migration Status	Population Background	CASP Score
Ahmed (2008) [20]	Interviews; self-completion questionnaire	United Kingdom	High income	Permitted on broad social or economic grounds *	Decision making regarding prenatal testing and termination for genetic conditions between Pakistani and white European mothers	10 (19 in total study)	Migrants: first generation (*n* = 5), second generation (*n* = 5)	Pakistani	8
Arnot (2017) [21]	Interviews	Thailand	Middle income	Restricted at time of publishing (to preserve health/social economic grounds); currently permitted on request	Experiences with safe abortion referral program	14	Cross-border **, refugees, migrants	Burmese	9
Asnong (2018) [22]	Interviews; focus group discussions	Mae La Refugee Camp, Mae Ker Thai clinic: Thailand-Burma border	Middle income	Restricted at time of publishing; currently permitted on request	Refugee and migrant adolescents’ perceptions and experiences of pregnancy	20 female (pregnant adolescents); 20 male (husbands of pregnant adolescents, adolescent boys, non-pregnant adolescent girls)	Refugees, migrants	Burmese	8
Belton and Whitaker (2007) [23]	Ethnography: interviews; focus group discussions; free-list activities	Tak Province, Thailand	Middle income	Restricted at time of publishing; currently permitted on request	Barriers to contraceptive access; motivation and means for termination	43 inpatients with post-abortion complications, 10 male partners, 10 health workers, 20 community members	Migrants (women post-abortion, partners, community members and lay midwives)	Burmese	8
Belton (2007) [24]	Ethnography: interviews; focus group discussions; free-list activities	Tak Province, Thailand	Middle income	Restricted at time of publishing; currently permitted on request	Barriers in contraceptive access; traditional techniques to terminate pregnancy	43 inpatients with post-abortion complications, 10 male partners, 10 health workers, 20 community members	Migrants	Burmese	8
Botfield (2020) [11]	Interviews	Sydney, Australia	High income	Permitted on request	Migrant and refugee youth experiences and perspectives on unintended pregnancy and abortion	27	Refugees, migrants	Mixed: East and Southeast Asian, African, South American, Mediterranean, Middle-Eastern	9
Böttcher (2019) [25]	Focus group discussions	Gaza strip	Middle income	Permitted to save the pregnant person’s life	Causes and consequences of unintended pregnancy	21	Refugees	Palestinian	8
Deeb-Sossa (2014) [26]	Ethnography: participant observation; interviews	North Carolina, United States	High income	Legal at time of publishing; 12-week restriction from July 2023	Barriers to abortion access	12	Migrants	Mexican	9
Dhar (2017) [27]	Interviews	Philadelphia, Pennsylvania, United States	High income	On request at time of publishing; currently accessible, with restrictions and no protections	Sexual and reproductive health attitudes and beliefs of unmarried, young Bhutanese women	14	Refugees	Bhutanese	8
Fordyce (2012) [28]	Interviews; ethnographic	South Florida, United States	High income	On request at time of publishing; currently protected, with restrictions	Family planning; unintended pregnancy	27	Migrants	Haitian	8
Gedeon (2016) [29]	Interviews	Tak Province, Thailand	Middle income	Restricted at time of publishing; currently permitted on request, gestational limit 20 weeks	Barriers to reproductive healthcare; sexual and reproductive decision making	31	Refugees, migrants	Burmese	9
Gitsels-van der Wal (2014) [30]	Interviews	The Netherlands	High income	Permitted on request	Role of religion (Islam) on decision making regarding prenatal anomaly screening and termination	10	Migrants: first generation (*n* = 6), second generation (*n* = 4)	Turkish	9
Gitsels-van der Wal (2015) [31]	Interviews	The Netherlands	High income	Permitted on request	Role of religion (Islam) on decision making regarding prenatal anomaly screening and termination	12	Migrants: first generation (*n* = 6), second generation (*n* = 6)	Moroccan	8
Hegde (2012) [32]	Ethnography: interviews, semi-structured questionnaires	Thai-Cambodia border	Middle income	Restricted at time of publishing; Currently permitted on request, gestational limit 20 weeks	attitudes and practices towards unsafe abortions; abortion as contraceptive method	10 interviewees; 15 questionnaire respondents (30 questionnaire participants in total)	Migrants/cross-border **	Cambodian	7
Hounnaklang (2021) [33]	Observation, field notes, in-depth interviews	Surat Thani province, Thailand	Middle income	Permitted on request, gestational limit 20 week	Sexual and reproductive health attitudes and beliefs; practices	22	Migrants	Myanmar women	8
Khin (2021) [34]	Interviews	Japan	High income	Permitted on broad social or economic grounds	Access to reproductive healthcare	17	Mixed residency status, including dependents, work visas, permanent/long-term residents	Myanmar women	8
Liamputtong (2003) [35]	In-depth interviews, participant observation	Melbourne, Australia	High income	Permitted on request; available but criminal at time of publishing; decriminalised 2008	Cultural practices and beliefs regarding abortion	27	Refugees, residing in Australia for 1–10+ years; spent minimum 1 year in Thai refugee camp	Hmong women	8
Nara (2019) [36]	Interviews; focus group discussions (FGDs)	Kampala and the Nakivale Refugee Settlement, Uganda	Low income	Permitted to save the pregnant person’s life	Reproductive healthcare; contraception and abortion/post-abortion services	21 interviewees; 36 in FGDs	Refugees	Congolese women	9
Ostrach (2020) [37]	Interviews; rapid ethnographic assessment	Catalunya, Spain	High income	Permitted on request	Experiences with legal, publicly funded abortion	13 (28 total participants)	Migrants	Not provided	9
Puri (2011) [38]	Interviews	California, New York, New Jersey, The United States	High income	Permitted on request at time of publishing; currently protected	Sex-selective abortion practices and experiences	65	Migrants	Indian women; Sikh (65%), Hindu (22%), (12%) Muslim (1%)	7
Remennick (2001) [39]	Interviews	Israel	High income	Permitted preserve health	Abortion experiences of native Israelis and recent Russian immigrants	25 (48 total participants)	Recent migrants	Russian women (former Soviet Union)	9
Rocha (2013) [40]	Focus group discussions and demographic questionnaire	Portugal	High income	Permitted on request	Sexual and reproductive health; maternity, pregnancy, induced abortion	35	Migrants	Brazil and Portuguese-speaking African countries (Lusophone): 15 Brazilians, 20 Africans	7
Royer (2020) [41]	Focus group discussions	The United States	High income	Permitted on request at time of publishing; currently dependent on state law	Family planning knowledge, attitudes, and practices	66	Refugees	Somali and Congolese women	10
Schoevers (2010) [42]	Semi-structured interviews	The Netherlands	High income	Permitted on request	Sexual and reproductive health problems and needs	100	Illegal immigrants	Mixed: Eastern Europe, Yugoslavia, former USSR; Middle East and North Africa; China, Mongolia; South America; Philippines; Surinam	8
Tousaw (2017) [43]	Interviews	Mae Sot, Thailand	Middle income	Restricted at time of publishing; currently permitted on request, gestational limit 20 weeks	Experiences of and perceptions on Safe Abortion Referral Program (SARP)	22	Documented (*n* = 10) and undocumented (*n* = 12) migrants	Burmese	9
Tousaw (2018) [44]	Interviews	Thailand-Burma border	Middle income	Restricted at time of publishing; currently permitted on request, gestational limit 20 weeks	Experiences of and perspectives on community-based misoprostol program	16	Cross-border, refugees, migrants	Burmese	9
Tucker (2015) [45]	Interviews	The United States	High income	Permitted on request at time of publishing; currently dependent on state law	Motivations for sex-selective abortions	20	Migrants	Indian	8
Udmuangpia (2017) [46]	Focus group discussions	Sweden	High income	Permitted on request	Perspectives on sexual behaviour and pregnancy	18	Adolescent migrants	Thai	9

* Colour coding follows the Center for Reproductive Rights classification scheme. Abortion legality is categorised as per the Center for Reproductive Rights’ five levels of legal permissibility, from least to most restrictive: On request, broad social or economic grounds, to preserve health, to save pregnant person’s life, prohibited altogether [47]; ** Cross-border migrants refer to those who migrate temporarily for work across borders; participants are not necessarily living as migrants at the time of the study but spend substantial time moving across borders.

## References

[1] Bearak J., Popinchalk A., Ganatra B., Moller A.-B., Tunçalp Ö., Beavin C., Kwok L., Alkema L. (2020). Unintended pregnancy and abortion by income, region, and the legal status of abortion: Estimates from a comprehensive model for 1990–2019. Lancet Glob. Health.

[2] Bateson D., McNamee K., Harvey C. (2021). Medical abortion in primary care. Aust. Prescr..

[3] World Health Organization (2022). Abortion Care Guideline.

[4] Coast E., Norris A.H., Moore A.M., Freeman E. (2018). Trajectories of women’s abortion-related care: A conceptual framework. Social. Sci. Med..

[5] Ross L., Solinger R. (2017). Reproductive Justice: An Introduction.

[6] Metusela C., Ussher J., Perz J., Hawkey A., Morrow M., Narchal R., Estoesta J., Monteiro M. (2017). “In My Culture, We Don’t Know Anything About That”: Sexual and Reproductive Health of Migrant and Refugee Women. Int. J. Behav. Med..

[7] Napier-Raman S., Hossain S.Z., Lee M.-J., Mpofu E., Liamputtong P., Dune T. (2023). Migrant and Refugee Youth Perspectives On Sexual and Reproductive Health and Rights in Australia: A Systematic Review. Sex. Health.

[8] Botfield J.R., Newman C.E., Kang M., Zwi A.B. (2018). Talking to migrant and refugee young people about sexual health in general practice. Aust. J. General. Pract..

[9] Mengesha Z., Perz J., Dune T., Ussher J. (2017). Refugee and migrant women’s engagement with sexual and reproductive health care in Australia: A socio-ecological analysis of health care professional perspectives. PLoS ONE.

[10] Ussher J.M., Rhyder-Obid M., Perz J., Rae M., Wong T.W.K., Newman P. (2012). Purity, Privacy and Procreation: Constructions and Experiences of Sexual and Reproductive Health in Assyrian and Karen Women Living in Australia. Sex. Cult..

[11] Botfield J.R., Newman C.E., Bateson D., Haire B., Estoesta J., Forster C., Schulz Moore J. (2020). Young migrant and refugee people’s views on unintended pregnancy and abortion in Sydney. Health Sociol. Rev..

[12] Agbemenu K., Hannan M., Kitutu J., Terry M.A., Doswell W. (2018). “Sex Will Make Your Fingers Grow Thin and Then You Die”: The Interplay of Culture, Myths, and Taboos on African Immigrant Mothers’ Perceptions of Reproductive Health Education with Their Daughters Aged 10-14 Years. J. Immigr. Minor. Health.

[13] Wanigaratne S., Wiedmeyer M.L., Brown H.K., Guttmann A., Urquia M.L. (2020). Induced abortion according to immigrants’ birthplace: A population-based cohort study. Reprod. Health.

[14] Chae S., Desai S., Crowell M., Sedgh G. (2017). Reasons why women have induced abortions: A synthesis of findings from 14 countries. Contraception.

[15] Lie M.L.S., Robson S.C., May C.R. (2008). Experiences of abortion: A narrative review of qualitative studies. BMC Health Serv. Res..

[16] Kirkman M., Rowe H., Hardiman A., Mallett S., Rosenthal D. (2009). Reasons women give for abortion: A review of the literature. Arch. Women’s Ment. Health.

[17] The EndNote Team (2013). EndNote.

[18] Page M.J., McKenzie J.E., Bossuyt P.M., Boutron I., Hoffmann T.C., Mulrow C.D., Shamseer L., Tetzlaff J.M., Akl E.A., Brennan S.E. (2021). The PRISMA 2020 statement: An updated guideline for reporting systematic reviews. BMJ.

[19] Critical Appraisal Skills Programme (2018). CASP (Qualitative) Checklist [Online]. https://casp-uk.net/casp-tools-checklists/.

[20] Ahmed S., Hewison J., Green J.M., Cuckle H.S., Hirst J., Thornton J.G. (2008). Decisions about testing and termination of pregnancy for different fetal conditions: A qualitative study of European White and Pakistani mothers of affected children. J. Genet. Couns..

[21] Arnott G., Tho E., Guroong N., Foster A.M. (2017). To be, or not to be, referred: A qualitative study of women from Burma’s access to legal abortion care in Thailand. PLoS ONE.

[22] Asnong C., Fellmeth G., Plugge E., Wai N.S., Pimanpanarak M., Paw M.K., Charunwatthana P., Nosten F., McGready R. (2018). Adolescents’ perceptions and experiences of pregnancy in refugee and migrant communities on the Thailand-Myanmar border: A qualitative study. Reprod. Health.

[23] Belton S., Whittaker A. (2007). Kathy Pan, sticks and pummelling: Techniques used to induce abortion by Burmese women on the Thai border. Soc. Sci. Med..

[24] Belton S. (2007). Borders of fertility: Unplanned pregnancy and unsafe abortion in Burmese women migrating to Thailand. Health Care Women Int..

[25] Böttcher B., Abu-El-Noor M.A., Nasser Ibrahim A.-E.-N. (2019). Causes and consequences of unintended pregnancies in the Gaza Strip: A qualitative study. BMJ Sex. Reprod. Health.

[26] Deeb-Sossa N., Billings D.L. (2014). Barriers to abortion facing Mexican immigrants in North Carolina: Choosing folk healers versus standard medical options. Lat. Stud..

[27] Dhar C.P., Kaflay D., Dowshen N., Miller V.A., Ginsburg K.R., Barg F.K., Yun K. (2017). Attitudes and Beliefs Pertaining to Sexual and Reproductive Health Among Unmarried, Female Bhutanese Refugee Youth in Philadelphia. J. Adolesc. Health.

[28] Fordyce L. (2012). Responsible Choices: Situating Pregnancy Intention among Haitians in South Florida. Med. Anthropol. Q..

[29] Gedeon J., Hsue S., Foster A. (2016). “I came by the bicycle so we can avoid the police”: Factors shaping reproductive health decision-making on the Thailand-Burma border. Int. J. Popul. Stud..

[30] Gitsels-van der Wal J.T., Manniën J., Ghaly M.M., Verhoeven P.S., Hutton E.K., Reinders H.S. (2014). The role of religion in decision-making on antenatal screening of congenital anomalies: A qualitative study amongst Muslim Turkish origin immigrants. Midwifery.

[31] Gitsels-van der Wal J.T., Martin L., Manniën J., Verhoeven P., Hutton E.K., Reinders H.S. (2015). A qualitative study on how Muslim women of Moroccan descent approach antenatal anomaly screening. Midwifery.

[32] Hegde S., Hoban E., Nevill A. (2012). Unsafe abortion as a birth control method: Maternal mortality risks among unmarried Cambodian migrant women on the Thai-Cambodia border. Asia-Pac. J. Public Health.

[33] Hounnaklang N., Sarnkhaowkhom C., Bannatham R. (2021). The beliefs and practices on sexual health and sexual transmitted infection prevention of Myanmar migrant workers in Thailand. Open Public Health J..

[34] Khin Y.P., Nawa N., Fujiwara T., Surkan P.J. (2021). Access to contraceptive services among Myanmar women living in Japan: A qualitative study. Contraception.

[35] Liamputtong P. (2003). Abortion--it is for some women only! Hmong women’s perceptions of abortion. Health Care Women Int..

[36] Nara R., Banura A., Foster A.M. (2019). Exploring Congolese refugees’ experiences with abortion care in Uganda: A multi-methods qualitative study. Sex. Reprod. Health Matters.

[37] Ostrach B. (2020). Publicly funded abortion and marginalised people’s experiences in Catalunya: A longitudinal, comparative study. Anthropol. Action..

[38] Puri S., Adams V., Ivey S., Nachtigall R.D. (2011). “There is such a thing as too many daughters, but not too many sons”: A qualitative study of son preference and fetal sex selection among Indian immigrants in the United States. Soc. Sci. Med..

[39] Remennick L.I., Segal R. (2001). Socio-cultural context and women’s experiences of abortion: Israeli women and Russian immigrants compared. Cult. Health Sex..

[40] Rocha C.M.F., Darsie C., da Silva V.C., Koetz A.P.M., Gama A.F., Dias S.F. (2013). Displaced maternity: Pregnancy, voluntary abortion and women’s health for immigrant women in Portugal. Rev. Bras. Em Promocao Da Saude.

[41] Royer P.A., Olson L.M., Jackson B., Weber L.S., Gawron L., Sanders J.N., Turok D.K. (2020). “In Africa, There Was No Family Planning. Every Year You Just Give Birth”: Family Planning Knowledge, Attitudes, and Practices Among Somali and Congolese Refugee Women after Resettlement to the United States. Qual. Health Res..

[42] Schoevers M.A., van den Muijsenbergh M.E., Lagro-Janssen A.L. (2010). Illegal female immigrants in The Netherlands have unmet needs in sexual and reproductive health. J. Psychosom. Obstet. Gynecol..

[43] Tousaw E., La R.K., Arnott G., Chinthakanan O., Foster A.M. (2017). “Without this program, women can lose their lives”: Migrant women’s experiences with the Safe Abortion Referral Programme in Chiang Mai, Thailand. Reprod. Health Matters.

[44] Tousaw E., Moo S., Arnott G., Foster A.M. (2018). “It is just like having a period with back pain”: Exploring women’s experiences with community-based distribution of misoprostol for early abortion on the Thailand-Burma border. Contraception.

[45] Tucker J., Moon S.S., Kim M., Kim K.S. (2015). The study on the motivation of sex-selective abortion among Indian immigrants in U.S.A. Int. J. Bio-Sci. Bio-Technol..

[46] Udmuangpia T., Haggstrom-Nordin E., Worawong C., Tanglakmankhonge K., Bloom T. (2017). A Qualitative study: Perceptions Regarding Adolescent Pregnancy Among A Group of Thai Adolescents in Sweden. Pac. Rim Int. J. Nurs. Res..

[47] Center for Reproductive Rights (2023). The World’s Abortion Laws. https://reproductiverights.org/maps/worlds-abortion-laws/.

[48] Thomas J., Harden A. (2008). Methods for the thematic synthesis of qualitative research in systematic reviews. BMC Med. Res. Methodol..

[49] (2020). NVivo.

[50] The World Bank WDI—The World by Income and Region. https://datatopics.worldbank.org/world-development-indicators/the-world-by-income-and-region.html.

[51] Wray A., Ussher J.M., Perz J. (2014). Constructions and experiences of sexual health among young, heterosexual, unmarried Muslim women immigrants in Australia. Cult. Health Sex..

[52] Hanschmidt F., Linde K., Hilbert A., Riedel- Heller S.G., Kersting A. (2016). Abortion Stigma: A Systematic Review. Perspect. Sex. Reprod. Health.

[53] Lokubal P., Corcuera I., Balil J.M., Frischer S.R., Kayemba C.N., Kurinczuk J.J., Opondo C., Nair M. (2022). Abortion decision-making process trajectories and determinants in low—And middle-income countries: A mixed-methods systematic review and meta-analysis. EClinicalMedicine.

[54] Rehnström Loi U., Lindgren M., Faxelid E., Oguttu M., Klingberg-Allvin M. (2018). Decision-making preceding induced abortion: A qualitative study of women’s experiences in Kisumu, Kenya. Reprod. Health.

[55] Pagoto S.L., Palmer L., Horwitz-Willis N. (2023). The Next Infodemic: Abortion Misinformation. J. Med. Internet Res..

[56] Assifi A.R., Berger B., Tunçalp Ö., Khosla R., Ganatra B. (2016). Women’s Awareness and Knowledge of Abortion Laws: A Systematic Review. PLoS ONE.

[57] Casey S.E., Isa G.P., Isumbisho Mazambi E., Giuffrida M.M., Jayne Kulkarni M., Perera S.M. (2022). Community perceptions of the impact of war on unintended pregnancy and induced abortion in Protection of Civilian sites in Juba, South Sudan. Glob. Public Health.

[58] Casey S.E., Steven V.J., Deitch J., Dumas E.F., Gallagher M.C., Martinez S., Morris C.N., Rafanoharana R.V., Wheeler E. (2019). “You must first save her life”: Community perceptions towards induced abortion and post-abortion care in North and South Kivu, Democratic Republic of the Congo. Sex. Reprod. Health Matters.

